# Epidemiology of Infections Caused by Gram-Positive Cocci—*Staphylococcus aureus*, *Streptococcus pneumoniae* and *Enterococcus faecium*: Antibiotic Resistance and Future Prospects

**DOI:** 10.3390/microorganisms14091951

**Published:** 2026-09-03

**Authors:** Victoria Birlutiu, Rares-Mircea Birlutiu

**Affiliations:** 1 Faculty of Medicine, Lucian Blaga University of Sibiu, Str. Lucian Blaga, Nr. 2A, 550169 Sibiu, Romania; victoria.birlutiu@ulbsibiu.ro; 2 County Clinical Emergency Hospital, Bvd Corneliu Coposu, Nr. 2–4, 550245 Sibiu, Romania; 3 Faculty of Medicine, The “Carol Davila” University of Medicine and Pharmacy, 050474 Bucharest, Romania; 4 “Foisor” Clinical Hospital of Orthopaedics, Traumatology and Osteoarticular TB, 021382 Bucharest, Romania

**Keywords:** antimicrobial resistance, Gram-positive cocci, *Staphylococcus aureus*, MRSA, *Streptococcus pneumoniae*, *Enterococcus faecium*, resistance mechanisms, novel antibacterial agents

## Abstract

Antimicrobial resistance is now documented in a substantial proportion of laboratory-confirmed bacterial infections worldwide, and although the most extreme extensively drug-resistant (XDR) and pandrug-resistant (PDR) phenotypes are concentrated among Gram-negative bacilli, resistance in Gram-positive cocci continues to rise and remains a major determinant of outcomes in severe infection. This structured comprehensive narrative review, based on a documented search of PubMed/MEDLINE, Scopus and Web of Science to June 2026, is confined to the three Gram-positive cocci of greatest clinical importance—*Staphylococcus aureus*, *Streptococcus pneumoniae* and *Enterococcus faecium*—and summarises current global and European surveillance data for this organisms, describes the principal molecular mechanisms responsible for resistance to β-lactams, glycopeptides, fluoroquinolones, oxazolidinones, lipopeptides, tetracyclines, folate-pathway inhibitors, macrolides–lincosamides–streptogramins and aminoglycosides, and reviews the therapeutic options and investigational agents that may extend the treatment repertoire against multidrug-resistant (MDR) Gram-positive cocci. Surveillance indicators for the three species are compared side by side, and the agents discussed are separated according to whether they are licensed, clinically evaluated but not widely licensed, or still preclinical. Its scope is deliberately narrower than that of pan-bacterial accounts of antimicrobial resistance: it concentrates on the determinants that routine molecular confirmation does not detect, on the divergent pathogen-specific trajectories visible in recent surveillance, and on the distinction between agents supported by randomised evidence and those still at the discovery stage. Our review is structured rather than systematic; the PRISMA 2020 flow diagram is used as a reporting template only, and no claim of PRISMA compliance is made.

## 1. Introduction

Data published by the World Health Organization (WHO) show that, between 2018 and 2023, resistance increased in more than 40% of the pathogen–antibiotic combinations under global surveillance, corresponding to an average annual increase of 5–15% [1]. The most severely affected areas are the South-East Asia and Eastern Mediterranean regions, where approximately one in three reported infections is resistant to the antibiotics used to treat it [1]. In 2023, one in six laboratory-confirmed bacterial infections worldwide was caused by an antibiotic-resistant organism. Among the infections monitored by GLASS (the Global Antimicrobial Resistance and Use Surveillance System), median resistance was highest in urinary tract infections (approximately one in three) and bloodstream infections (one in six), and lower in gastrointestinal infections (one in 15) and urogenital gonococcal infections (one in 125) [1]. In the regions most affected by resistance, one in three infections in South-East Asia and the Eastern Mediterranean is caused by resistant bacteria, compared with one in five infections in the African Region, one in ten in the European Region and one in 11 in the Western Pacific Region. Whereas the global median resistance is 17.2%, it stands at 31.1% in the South-East Asia Region and at 30.0% in the Eastern Mediterranean Region [1].

Countries with underfunded health systems and low per capita income are evidently more frequently associated with elevated levels of antibiotic resistance among isolated microorganisms, which further complicates their treatment with new, costly antibiotics. Despite these differences in geography, infrastructure, and the control of infection and of antibiotic administration, *E. coli*, *K. pneumoniae*, and *S. aureus*—including MRSA—predominate globally in bloodstream infections, in both community-acquired and nosocomial infections, whereas *Salmonella* and *S. pneumoniae* are encountered predominantly in community-acquired systemic infections, according to GLASS. In 2023, *E. coli* accounted for 44.9% of blood culture isolates worldwide, while *S. aureus* was responsible for 21.9% of identifications, *K. pneumoniae* for 21.1%, *Acinetobacter* for 7.2%, *S. pneumoniae* for 1.4%, and *Salmonella* spp. for 1.4% [1].

With regard to *S. aureus* resistance in 2023, 27.1% of the isolated strains were MRSA worldwide, with values differing in relation to economic level and AMR surveillance: 9.7% in European countries, with the exception of the Mediterranean region, where the reported resistance rate was 50.3%. For *S. pneumoniae*, the global level of resistance was 17.8% to oxacillin (a surrogate for strains with intermediate susceptibility to penicillin) and 5.2% to penicillin; resistance to third-generation cephalosporins remains low, at 0.7% for ceftriaxone and 0.8% for cefotaxime [1].

Against this background, integrated measures are required in all countries: prevention and control; bacteriological identification in laboratories equipped for rapid detection, including detection of the underlying resistance mechanisms; antimicrobial stewardship; implementation of national action plans; sanitation, hygiene and vaccination measures; and control of antibiotic use in animal husbandry, among others.

Gram-positive cocci account for a substantial share of that burden, and the trajectory of resistance within this group is neither uniform across organisms nor uniform across regions. This review examines three species of major clinical importance—*Staphylococcus aureus*, *Streptococcus pneumoniae* and *Enterococcus faecium*—with three objectives: to summarise what recent global and European surveillance shows about the burden and direction of resistance in each; to describe the molecular determinants responsible, with attention to those that routine laboratory confirmation does not detect; and to appraise the therapeutic repertoire that remains, distinguishing agents supported by randomised clinical evidence from those still at the discovery stage. Other Gram-positive cocci of clinical relevance, including coagulase-negative staphylococci, *Enterococcus faecalis* and the pyogenic streptococci, fall outside this scope and are not considered in this review.

The epidemiology of multidrug resistance across the bacterial spectrum, together with the general mechanisms that underlie it, has recently been reviewed by us [2]. That review was deliberately broad: it encompassed Gram-negative and Gram-positive organisms and the generic mechanistic categories common to both, and its therapeutic sections were weighted towards carbapenem-resistant Gram-negative bacilli. The present review narrows the field of view to the three Gram-positive cocci that carry the greatest clinical burden and examines them at a level of detail that a pan-bacterial account cannot accommodate: pathogen-specific data from the most recent global and European reporting cycles, the *mec* homologues and transferable oxazolidinone determinants that escape routine molecular confirmation, the modification of pneumococcal resistance by conjugate vaccination, and a therapeutic appraisal that separates agents supported by randomised trials from those still preclinical. The two articles are complementary rather than overlapping, the earlier providing panoramic context and the present one the pathogen-level detail on which clinical and stewardship decisions in Gram-positive infection depend.

## 2. Materials and Methods

### 2.1. Type of Review and Aim

This article is a structured comprehensive narrative review. The literature was identified through an explicit, dated and documented search, and eligibility was governed by pre-specified criteria, but the synthesis is interpretative rather than systematic: no protocol was registered, screening was not conducted in duplicate with formal adjudication, no structured extraction instrument was used, and no risk-of-bias appraisal was undertaken.

This design was adopted deliberately rather than by default. The three questions addressed here—how the epidemiology of resistance in *Staphylococcus aureus*, *Streptococcus pneumoniae* and *Enterococcus faecium* has changed across recent surveillance cycles, which molecular determinants account for that resistance, and which therapeutic options remain or are emerging—draw on evidence of fundamentally different kinds, including population-based surveillance reports, molecular microbiological studies, randomised controlled trials and preclinical discovery work. Such evidence cannot be pooled quantitatively, and no single quality-appraisal instrument applies meaningfully across it. The review therefore aims to integrate rather than to aggregate, while retaining the reporting transparency expected of a documented search. Because the work drew exclusively on previously published literature, it involved no human participants, biological specimens or individual patient data, and no primary statistical analysis was undertaken.

**Figure 1:**
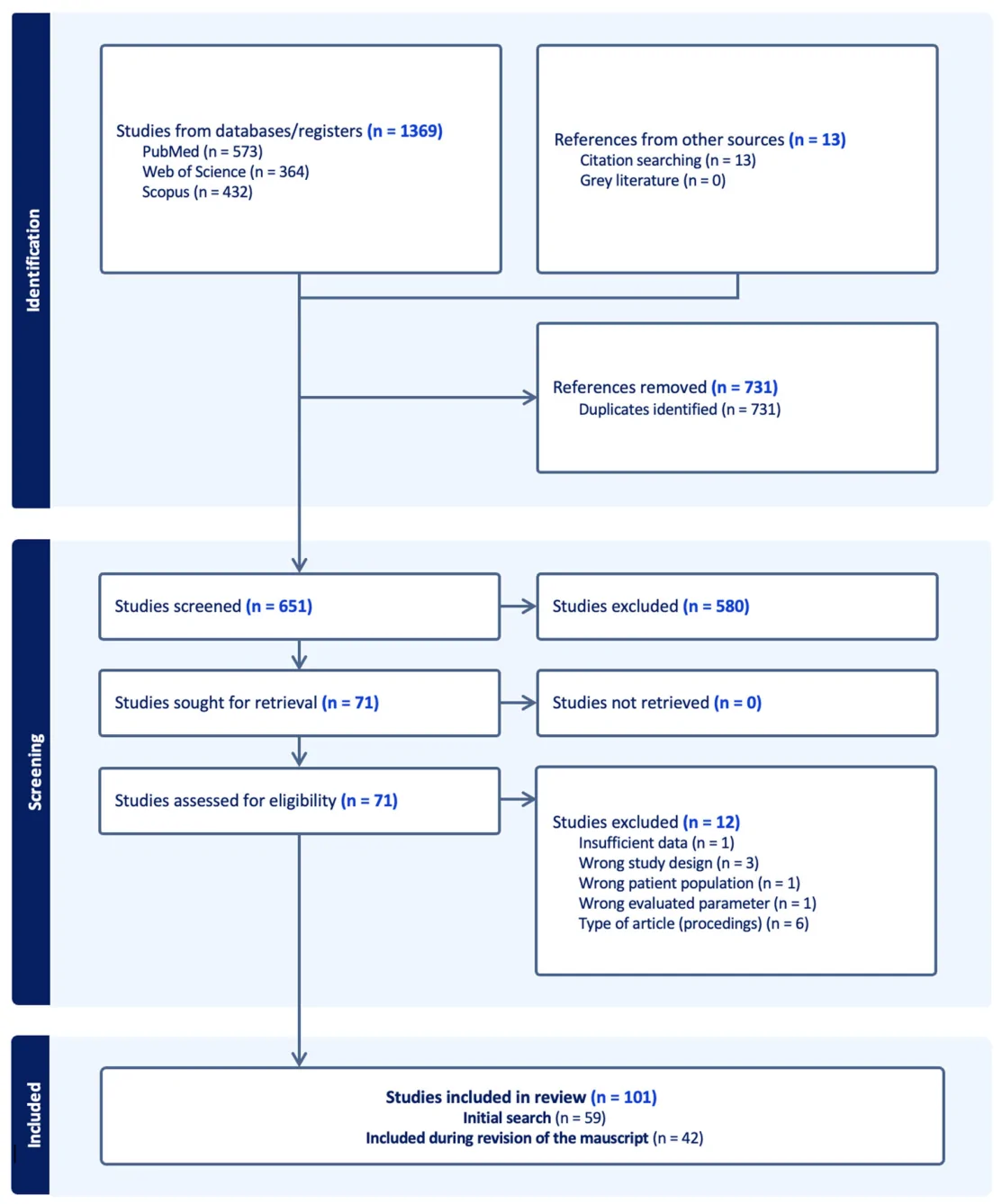
Flow of records through identification, screening and inclusion. The diagram follows the layout of the PRISMA 2020 flow diagram, which was used solely as a reporting template for the flow of records. The present article is a structured comprehensive narrative review and does not claim compliance with the PRISMA 2020 statement.

Because the flow of records is reported using the layout of the PRISMA 2020 flow diagram (Figure 1), the distinction between the present design and a systematic review is stated here explicitly. PRISMA 2020 [3] was adopted solely as a reporting template for the number of records identified, screened, excluded and retained; it was not applied as a methodological standard, no PRISMA checklist accompanies this article, and no claim of PRISMA compliance is made. Table 1 sets out, element by element, which features of a systematic review were applied and which were not. The present review addresses three related questions that draw on evidence of incompatible types and integrates them interpretatively. Reporting the flow of records transparently improves the accountability of a narrative synthesis; it does not convert it into a systematic review, and it is not presented as doing so.

### 2.2. Data Sources and Search Strategy

Three bibliographic databases, PubMed/MEDLINE, Scopus and Web of Science, were searched separately, each through its own interface and with its own syntax, and the results were subsequently combined; no federated or single-platform search was used. Coverage extended to publications from 1990 onwards, and the final search was executed in each database on 1 June 2026. The search was restricted to articles published in English. Two complementary approaches were used within each database. A core Boolean query, reproduced below, was developed in PubMed/MEDLINE and translated into the equivalent field syntax of the other two platforms. In addition, combinations of free-text keywords and their synonyms were used, including terms such as “antimicrobial resistance”, “multidrug resistance”, “Gram-positive cocci”, “*Staphylococcus aureus*”, “MRSA”, “methicillin resistance”, “mecA”, “vancomycin-intermediate and vancomycin-resistant S. aureus”, “*Streptococcus pneumoniae*”, “penicillin resistance”, “macrolide resistance”, “*Enterococcus faecium*”, “vancomycin resistance” and “novel antibacterial agents”, and searched separately from the core query in order to retrieve records that the latter did not return.

The following search string was used in PubMed/MEDLINE and adapted to Scopus and Web of Science. The searches were restricted to English-language publications, and the last search was run on 1 June 2026. (“antimicrobial resistance” [Mesh] OR “antimicrobial resistance” [tiab] OR “antibiotic resistance” [tiab] OR “multidrug resistance” [tiab] OR “multidrug-resistant” [tiab] OR MDR [tiab] OR XDR [tiab] OR PDR [tiab] OR “methicillin resistance” [tiab] OR “penicillin resistance” [tiab] OR “macrolide resistance” [tiab] OR “vancomycin resistance” [tiab]) AND (“resistance mechanisms” [tiab] OR “enzymatic degradation” [tiab] OR “target modification” [tiab] OR “efflux pump” [tiab] OR “biofilm” [tiab] OR mecA [tiab]) AND (“Gram-positive cocci” [tiab] OR “Staphylococcus aureus” [tiab] OR MRSA [tiab] OR “vancomycin-intermediate Staphylococcus aureus” [tiab] OR “vancomycin-resistant Staphylococcus aureus” [tiab] OR “Streptococcus pneumoniae” [tiab] OR “Enterococcus faecium” [tiab]) AND (“novel antibiotics” [tiab] OR “novel antibacterial agents” [tiab] OR “emerging therapies” [tiab] OR “alternative therapies” [tiab] OR “bacteriophage” [tiab] OR “peptide antibiotic” [tiab]) AND (1990:2026 [pdat]) AND English [la] NOT (animals [mh] NOT humans [mh]). Equivalent Boolean queries were constructed for Scopus using the TITLE-ABS-KEY field and for Web of Science using the TS (Topic) field. The publication window was limited to 1990–2026 in all three databases.

The construction of the core query requires comment, since it determines what the search could and could not retrieve. Four concept blocks—resistance terminology, mechanism terminology, organism terminology and novel-agent terminology—are combined with the AND operator, so that only records addressing all four concepts simultaneously are returned. The query is therefore specific rather than sensitive: a surveillance report describing the epidemiology of MRSA alone, or a molecular study describing a single mechanism without reference to therapeutic development, does not satisfy all four blocks and is not retrieved by it. This was not the sole route of retrieval. The free-text keyword combinations listed above were searched independently of the core query, and the database searches were supplemented by the institutional sources and reference-list screening described below. Retrieval was nevertheless not exhaustive, and the consequences of this design for the completeness of the evidence base are acknowledged.

The database search was complemented by consultation of authoritative institutional sources, including the World Health Organization (WHO), the European Centre for Disease Prevention and Control (ECDC) and the Centers for Disease Control and Prevention (CDC), and by screening the reference lists of retrieved articles to identify further relevant publications. One study published after the search cut-off [4], known as in-press work, was included during manuscript preparation.

Records retrieved from the three databases were exported and combined in a single reference library maintained in Mendeley Reference Manager (Elsevier), in which duplicate records were identified using the built-in duplicate-detection function and the result was verified manually against digital object identifier, first author, title, journal and year of publication. Of the 1382 records identified, 731 were removed as duplicates, leaving 651 unique records. Deduplication was the only operation carried out before screening. The 651 unique records then entered title-and-abstract screening, conducted as described in Section 2.3 of the manuscript, from which 71 records were taken forward for full-text assessment; the 580 records not taken forward are reported as screening exclusions rather than as removals preceding screening. The number of records included through the documented search (*n* = 59) is smaller than the number of references cited in this article, because authoritative institutional sources, methodological references and work identified after the search cut-off were cited without having entered the review through the database search, but these are counted in Figure 1.

### 2.3. Study Selection and Eligibility Criteria

Records were eligible if they reported peer-reviewed original clinical or microbiological data, systematic reviews or meta-analyses, randomised controlled trials, prospective or retrospective cohort studies, international guidelines or consensus statements, or official epidemiological reports, and if they bore directly on resistance in *S. aureus*, *S. pneumoniae* or *E. faecium*. Publications from the preceding five to ten years were preferred; older work was retained only where it established a mechanism, phenotype or definition still in current use. Conference abstracts without accessible full text, non-peer-reviewed material, publications from predatory outlets and non-authoritative internet sources were excluded, as were studies confined to fungal, viral or parasitic resistance, to veterinary pathogens without direct human relevance, or to Gram-negative organisms except where these were the documented origin of a determinant subsequently identified in Gram-positive cocci.

Titles and abstracts were screened by both authors, and full texts were retrieved for records that could not be excluded on the abstract alone.

### 2.4. Data Extraction and Synthesis

Information was extracted by both authors on the burden and distribution of resistance, the determinants and mechanisms responsible, and the licensed or investigational agents active against the organisms concerned. The evidence was synthesised qualitatively and organised first by pathogen and then, within each pathogen, by antibiotic class and corresponding mechanism; therapeutic evidence was organised separately according to regulatory and developmental status. No quantitative synthesis was undertaken, and, consistent with the design set out in Section 2.1, no formal risk-of-bias or quality-appraisal instrument was applied. Where sources reported conflicting findings, the discrepancy is presented as such rather than resolved.

### 2.5. Ethical Considerations

Ethics committee approval and informed consent were not required, since the review used only previously published data and involved neither human participants nor identifiable personal information nor animals. The primary studies cited remain covered by the approvals reported in their original publications.

The study selection process is presented in a flow diagram (Figure 1) similar to [3].

## 3. Results and Discussions

### 3.1. Gram-Positive Cocci in the Current Resistance Landscape

Although the most serious XDR and PDR problems are associated with infections caused by Gram-negative bacteria, Gram-positive cocci should not be underestimated, since they are responsible for severe infections and their resistance is following an upward global trend, in particular *Staphylococcus aureus*, *Streptococcus pneumoniae* and *Enterococcus faecium*. *S. aureus* was associated with one million deaths worldwide in 2019, with cases reported from 135 countries, predominantly in patients older than 15 years presenting with sepsis, whereas *S. pneumoniae* is associated with deaths mainly in children under 5 years of age and with lower respiratory tract infections [5]. In May 2024, the WHO retained methicillin-resistant *S. aureus* (MRSA) and vancomycin-resistant *E. faecium* in the high-priority category of its updated Bacterial Priority Pathogens List, alongside fluoroquinolone-resistant *Salmonella* Typhi, fluoroquinolone-resistant *Shigella* spp., carbapenem-resistant *Pseudomonas aeruginosa*, fluoroquinolone-resistant non-typhoidal *Salmonella*, and *Neisseria gonorrhoeae* resistant to third-generation cephalosporins and/or fluoroquinolones [6,7]. Within that prioritisation exercise, vancomycin-resistant *E. faecium* (69%) and MRSA (59%) obtained the highest overall scores among Gram-positive bacteria, while macrolide-resistant *S. pneumoniae* and macrolide-resistant group A streptococci were assigned to the medium-priority tier [7].

Current data are undoubtedly incomplete: in some countries, and particularly in sub-Saharan Africa, AMR surveillance is fragmented and regional in scope, and uneven monitoring may influence the true magnitude of antimicrobial resistance, the trends in its development and, not least, the specific interventions intended to limit this phenomenon [8].

The judicious use of antibiotics represents the principal measure capable of reducing the pressure driving the development of bacterial resistance. Ensuring that antibiotics from the Access group (penicillins, ampicillin, oxacillin, amoxicillin, ampicillin–sulbactam, amoxicillin–clavulanate, first-generation cephalosporins, clindamycin, sulfonamides, imidazoles, doxycycline, tetracycline) account for at least 70% of use in every country—agents with a narrow spectrum and a favourable safety profile—would significantly reduce bacterial resistance. The choice of Watch-group antibiotics (second-, third- and fourth-generation cephalosporins, aminoglycosides, carbapenems, fluoroquinolones, piperacillin–tazobactam, macrolides, lincomycin, rifamycins, fusidic acid, older glycopeptides—vancomycin, teicoplanin) as first-line therapy should be reserved for severe clinical presentations that may be caused by resistant organisms, with de-escalation as rapidly as possible once susceptibility results become available. The use of Reserve-group antibiotics (fifth-generation cephalosporins; carbapenem/β-lactamase inhibitor combinations; plazomicin; tigecycline; newer glycopeptides—dalbavancin, oritavancin, telavancin—monobactams; daptomycin; oxazolidinones; penems such as faropenem; intravenous fosfomycin; cefiderocol; polymyxins; pleuromutilins; streptogramins; newer intravenous tetracyclines; ceftazidime/avibactam; iclaprim) should be directed towards infections established with certainty to be caused by XDR, difficult-to-treat organisms, in accordance with susceptibility testing [9]. Antimicrobial stewardship programmes should be implemented nationally, in every country, as a strategic programme for the prevention or reduction in AMR. In the same vein, equipping microbiology laboratories should be the single most important action, enabling rapid identification of the species involved in the aetiology of systemic, respiratory, gastrointestinal and urinary infections, together with the determination of antibiotic susceptibility to support rational treatment.

Global modelling further projects that the number of deaths attributable to bacterial antimicrobial resistance will continue to rise until 2050 [10]. The wider bacterial context is set out in [2]; the present review is confined to Gram-positive cocci.

*S. aureus* is the bacterial agent most frequently responsible for sepsis, accounting for 10–30 cases per 100,000 inhabitants [4]. Multinational population-based surveillance covering 83 million person-years in Canada, Denmark, Finland, Sweden and Australia reported an overall age- and sex-standardised incidence of *S. aureus* bloodstream infection of 26.1 episodes per 100,000 population per year, a figure consistent with the range of 15–40 per 100,000 reported across population-based studies [11]. In 2023, 27.1% of strains isolated from blood cultures worldwide were MRSA [1], a proportion that reached 50.3% in the Eastern Mediterranean region.

European surveillance data illustrate that these global trends are not uniform. In the European Union/European Economic Area, the estimated incidence of MRSA bloodstream infection in 2024 was 4.48 per 100,000 population (country range 0.55–13.63), 20.4% lower than in the 2019 baseline year and already below the 2030 EU target of 4.79 per 100,000, with a statistically significant decreasing trend between 2019 and 2024. Over the same period, however, the estimated incidence of bloodstream infections caused by vancomycin-resistant *E. faecium* increased, and resistance levels remained highest in southern, central and eastern Europe [12]. Divergent trajectories of this kind indicate that infection prevention and stewardship interventions can modify the epidemiology of individual Gram-positive pathogens, and that surveillance data must be interpreted locally rather than extrapolated from global averages.

The surveillance picture is easier to read when the three organisms are placed side by side. Table 2 assembles the principal figures cited in this section. Two features deserve emphasis. First, the resistance marker that defines each organism is different, methicillin resistance in *S. aureus*, reduced susceptibility to penicillin in *S. pneumoniae*, vancomycin resistance in *E. faecium*, so that a single aggregate resistance proportion for “Gram-positive cocci” would have no meaning. Second, within the same surveillance system and over the same interval, the trajectories diverge: in the EU/EEA between 2019 and 2024, MRSA bloodstream infection fell significantly, while bloodstream infection caused by vancomycin-resistant *E. faecium* rose [12].

### 3.2. Resistance Mechanisms in Staphylococcus aureus

#### 3.2.1. Resistance to β-Lactams

Although methicillin resistance is the most frequent mechanism, inactivating the majority of β-lactams, the MODSA and BORSA phenotypes blur the boundary between susceptibility and resistance. These two phenotypes are not, however, mediated by *mec* genes: borderline oxacillin-resistant *S. aureus* (BORSA) isolates show oxacillin minimum inhibitory concentrations (MICs) of approximately 1–8 µg/mL and lack PBP2a encoded by *mecA* or *mecC*, their resistance being attributed to hyperproduction of β-lactamase or, in some isolates, to point mutations in native *pbp* genes, as in modified *S. aureus* (MODSA). Because they are neither truly methicillin-resistant nor truly methicillin-susceptible, such isolates are frequently misclassified, with potential therapeutic consequences [14]. MRSA was first isolated in the United Kingdom in 1961, and resistance is conferred by the production of PBP2a encoded by *mecA*, a protein with reduced affinity for β-lactams that was acquired from a heterologous source [15,16,17]. Comparative genomic analysis subsequently identified the animal-associated species *Staphylococcus fleurettii*, in which a chromosomal *mecA* gene is located among genes essential for staphylococcal growth and is not associated with a staphylococcal cassette chromosome *mec* (SCC*mec*) element, as the most probable origin and reservoir of the *mecA* gene [18]. In the 1990s, the emergence of community-associated MRSA (CA-MRSA) strains, distinct from healthcare-associated MRSA (HA-MRSA), was associated with resistance transmitted through transferable genomic islands, SCC*mec*, carrying *mecA* together with further genes conferring resistance to the lincosamide–macrolide–streptogramin group, to aminoglycosides, or to tetracyclines [19].

Two additional *mec* determinants deserve separate consideration, because they are not interchangeable with *mecA*. The divergent homologue *mecC* (originally designated *mecA*LGA251, sharing approximately 70% nucleotide identity with *mecA*) is carried on a distinct element, SCC*mec* type XI, and was first described in isolates from dairy cattle and from humans in the United Kingdom and Denmark; such isolates are phenotypically resistant but test negative in *mecA*-specific molecular assays [20]. By comparison, *mecB*, previously known only from *Macrococcus caseolyticus*, has been reported in *S. aureus* on an 84.6 kb transferable multidrug-resistance plasmid that also carries aminoglycoside, macrolide and tetracycline resistance genes, rather than within SCC*mec* [21]. Both mechanisms may escape detection by routine *mecA*-based confirmatory testing.

Beyond the production of PBP2a (PBP2′), staphylococcal resistance to β-lactams may also be associated with mutations in *pbp* genes (MODSA) or with the enzymatic synthesis of β-lactamases, which hydrolyse the β-lactam ring.

#### 3.2.2. Resistance to Glycopeptides

Vancomycin-intermediate *S. aureus* (VISA) strains, which are not inhibited in vitro by vancomycin concentrations of 4–8 µg/mL, were identified in Japan in 1996. This phenotype results from an increased concentration of non-cross-linked peptidoglycan chains within a thickened cell wall, whose D-Ala-D-Ala termini act as false targets and trap vancomycin in the outer layers of the wall [22,23].

Vancomycin-resistant *S. aureus* (VRSA) strains are defined by vancomycin MICs of at least 16 µg/mL, resistance having been acquired genetically from vancomycin-resistant enterococci (VRE). The *vanA* gene cluster directs synthesis of peptidoglycan precursors terminating in D-Ala-D-Lac, for which the affinity of vancomycin and teicoplanin is extremely low [15]. Three points of terminology should be distinguished, since they are frequently conflated. The VISA phenotype is not *van*-mediated: it arises from stepwise chromosomal mutation, most consistently affecting the *vraSR*, *walKR* (*yycFG*) and *graRS* regulatory systems and *rpoB*, and its expression depends on a thickened, poorly cross-linked wall rather than on any alteration of the D-Ala-D-Ala target itself [24]. Heterogeneous VISA (hVISA) describes an isolate that appears susceptible on routine minimum inhibitory concentration testing but contains subpopulations expressing the VISA phenotype at a frequency of the order of 10^−6^ to 10^−5^; it is not detected by standard susceptibility methods, requires population analysis profiling for confirmation, and is regarded as the state from which VISA is selected under glycopeptide exposure [24]. High-level resistance, by contrast, is target replacement and is transferable. Of the *van* gene clusters characterised in enterococci, only *vanA* has so far been reported in clinical VRSA isolates; *vanB*, *vanD*, *vanG* and *vanM* remain enterococcal, and *vanC* is a chromosomally encoded, intrinsic and low-level determinant of *Enterococcus gallinarum* and *Enterococcus casseliflavus* that is neither transferable nor associated with high-level resistance [24]. Describing these determinants collectively as reducing the binding affinity of glycopeptides, as earlier accounts have done, obscures the distinction between an intrinsic species marker and an acquired, mobile resistance operon [25,26].

#### 3.2.3. Resistance to Fluoroquinolones

Fluoroquinolone resistance is frequently associated with HA-MRSA strains and follows mutations in *the quinolone-resistance-determining region of gyrA,* the gene encoding the A subunit of DNA gyrase, the enzyme involved in bacterial DNA supercoiling, as well as alterations in *grlA*, which encodes a subunit of topoisomerase IV. The distinction is worth preserving: *gyrA* and *grlA* are genes, whereas GyrA and GrlA (ParC) are the protein subunits they encode, and resistance is conferred by amino-acid substitutions in the latter. Other mechanisms associated with fluoroquinolone resistance reduce permeability to intracellular antibiotic entry and enhance antibiotic elimination through overexpression of NorA efflux pumps [27].

#### 3.2.4. Resistance to Lincosamides, Streptogramins and Pleuromutilins

Methylation of the ribosomal target is not the only way in which staphylococci evade agents acting at the peptidyl transferase centre. A second route leaves both the drug and its binding site chemically unaltered: ABC-F proteins associate with the ribosome and protect it, displacing the bound antibiotic rather than modifying the site to which it binds. Sal-type ABC-F proteins have been characterised as intrinsic and widely distributed mediators of pleuromutilin resistance across staphylococcal species, with individual family members differing substantially in the range of antibiotics against which they confer protection; structural work places them in the ribosomal E site and indicates that displacement occurs allosterically rather than by direct competition at the drug binding site [28]. Two features give this mechanism practical significance. It is chromosomally encoded rather than acquired, so it is invisible to screening directed at transferable determinants; and it acts on the same ribosomal region as the mobile *optrA* and *poxtA* gene products which likewise encode ABC-F proteins and likewise confer resistance by target protection [29]. Target protection thus links an intrinsic staphylococcal trait to a class of transferable determinants of growing clinical concern.

#### 3.2.5. Resistance to Oxazolidinones and Lipopeptides

Linezolid resistance in MRSA is attributed to single-nucleotide mutations in the 23S rRNA gene, the site of action of the oxazolidinones [30]. In addition to these chromosomal mutations, seven transferable oxazolidinone resistance determinants have now been characterised: the *cfr* genes (*cfr*, *cfr*(B), *cfr*(C), *cfr*(D) and *cfr*(E)), which encode 23S rRNA methyltransferases conferring a multiresistance phenotype that includes phenicols, lincosamides, oxazolidinones, pleuromutilins and streptogramin A compounds, together with *optrA* and *poxtA*, which encode ABC-F proteins that protect the ribosome from oxazolidinones. These genes are most often plasmid-borne but also occur on transposons, integrative and conjugative elements, genomic islands and prophages, which allows dissemination across species and genus boundaries and permits co-selection by agents of other classes [29]. Their spread is clinically relevant because it may compromise linezolid in the treatment of MRSA and of vancomycin-resistant enterococci, and because it is not detected by resistance-gene panels restricted to 23S rRNA mutations.

In the case of daptomycin, an antibiotic recommended for the treatment of MRSA sepsis and endocarditis, reduced susceptibility is not conferred by any single determinant, and the term daptomycin-non-susceptible is preferable to daptomycin-resistant, since no resistance breakpoint is defined for this agent against *S. aureus*. The phenotype is selected stepwise and converges on cell-envelope homeostasis at three points. The first is gain-of-function mutation in *mprF*, which increases the synthesis and outer-leaflet translocation of lysyl-phosphatidylglycerol and thereby raises the net-positive charge of the membrane surface, repelling the calcium–daptomycin complex. The second is mutation in the *walKR* (*yycFG*) and *vraSR* regulatory systems, which alters cell-wall turnover and thickness and impedes access of the antibiotic to the membrane. The third is mutation in genes of phospholipid metabolism, including the cardiolipin synthase *cls2*, which redistributes the phosphatidylglycerol on which daptomycin binding depends [31]. Because several of these loci also contribute to reduced glycopeptide susceptibility, daptomycin non-susceptibility and the VISA phenotype are frequently encountered in the same isolate, and prior vancomycin exposure should raise suspicion of both [24,31,32].

#### 3.2.6. Resistance to Tetracyclines and to Folate-Pathway Inhibitors

The use of tetracyclines as antistaphylococcal therapy has allowed resistance to emerge by two mechanisms that should be kept distinct. The first is active efflux by major-facilitator-superfamily pumps encoded by the plasmid-borne genes *tet*(K) and *tet*(L). The second is ribosomal protection: *tet*(M) and *tet*(O) encode GTPases homologous to elongation factor G, which bind the ribosome and dislodge the drug from its binding site on the 30S subunit, with the resulting conformational change persisting long enough for translation to resume after the protein has dissociated [33]. Ribosomal protection is therefore not a modification of the target, and describing Tet(M) and Tet(O) as target-modifying enzymes, as the previous version of this section did, misstates the mechanism. The distinction has practical consequences: the glycylcycline tigecycline and the aminomethylcycline omadacycline are poor substrates for Tet(K) and Tet(L) and are not displaced from the ribosome by Tet(M) at clinically relevant concentrations, whereas the classical tetracyclines are affected by both mechanisms [34,35].

The sulfamethoxazole–trimethoprim combination may likewise be associated with the development of resistance, either to sulfamethoxazole through alterations in the chromosomal gene encoding dihydropteroate synthase (DHPS), or to trimethoprim through acquisition of the *dfrA* gene, which encodes an alternative target, dihydrofolate reductase (DHFR) [30,36,37].

#### 3.2.7. Resistance to Macrolides, Lincosamides, Streptogramins and Aminoglycosides

Clindamycin resistance is a consequence of *erm* genes, which encode ribosomal methyltransferases that methylate an adenine residue within domain V of the 23S rRNA subunit, thereby reducing the binding of macrolides, lincosamides and streptogramin B and producing the MLSB cross-resistance phenotype; this phenotype may be constitutive or inducible (iMLSB), with the latter being detectable only by double-disk diffusion (D-test) testing [15,38]. Aminoglycoside resistance is enzymatic in nature, mediated by the synthesis of acetyltransferases, phosphotransferases or nucleotidyltransferases that prevent binding to the 30S ribosomal target site, or by an efflux mechanism [39].

#### 3.2.8. Acquisition of Resistance Genes from Gram-Negative Bacilli

Class 1 integrons, genetic structures characteristic of Gram-negative bacteria, were identified in *S. aureus* in 2006, carrying gene cassettes associated with resistance to streptomycin (*aadA2*, *aadA5*), trimethoprim (*dfrA12*, *dfr17*) and chloramphenicol (*cmlA1*) [40,41,42]. These observations show that determinants of a type otherwise characteristic of Gram-negative organisms are present within the staphylococcal gene pool, and they are consistent with genetic exchange between the two groups. They do not, however, demonstrate direct transfer from a Gram-negative bacillus to *S. aureus*: the reports are observational, the direction of transfer was not established, and neither the route nor any intermediate host was identified experimentally. The finding is therefore presented here as an association compatible with interspecies exchange rather than as a confirmed mechanism. The best-documented acquisition of a resistance determinant by *S. aureus* from another species remains that of the *vanA* operon from vancomycin-resistant enterococci, a transfer between Gram-positive organisms [24].

First-line therapy in staphylococcal infections takes into account the level of resistance specific to the geographical region, namely the incidence of MRSA and VRSA and resistance to oxazolidinones, clindamycin, and so on. Depending on this, vancomycin or teicoplanin, and daptomycin, may be considered as first-line options until MRSA has been excluded, particularly in systemic infections. Oritavancin is an option in skin, soft-tissue, or osteoarticular infections, whereas telavancin is effective in cutaneous infections and in pneumonia [43]. Other antibiotics effective in staphylococcal infections include tigecycline (to be used with caution in systemic infections), ceftaroline, fusidic acid, and the fluoroquinolones; trimethoprim/sulfamethoxazole and rifampicin are therapeutic options depending on local resistance patterns [43].

### 3.3. Resistance in Streptococcus pneumoniae

*S. pneumoniae* occupies a different epidemiological position from the other two organisms considered here. It remains a leading cause of community-acquired pneumonia, bacterial meningitis and otitis media, and its burden falls disproportionately on children under five years of age and on older adults, in contrast to the predominantly adult and healthcare-associated burden of *S. aureus* and *E. faecium* [5]. Its numerical contribution to bloodstream infection is small—1.4% of blood-culture isolates reported to GLASS in 2023—but this understates its importance, since most pneumococcal disease is respiratory rather than bacteraemic and is therefore poorly captured by blood-culture-based surveillance [1]. Global resistance in 2023 stood at 17.8% to oxacillin, used as a surrogate for reduced penicillin susceptibility, and at 5.2% to penicillin, while resistance to third-generation cephalosporins remained below 1% [1]. The pneumococcus also differs from the staphylococci and the enterococci in the way resistance is generated. It is naturally transformable, and its resistance arises principally by homologous recombination with commensal streptococci of the mitis group rather than by the acquisition of discrete mobile resistance genes; the resulting mosaic alleles are stably maintained within a limited number of internationally disseminated multiresistant lineages [13,44,45].

In *S. pneumoniae*, penicillin resistance was identified as early as the 1960s and reflects modification of the penicillin-binding proteins (PBPs), the site of action of β-lactams, arising either from chromosomal mutations or from genetic material derived from other bacteria and incorporated into pneumococcal DNA; the acquisition of resistance has increased to a worrying degree, particularly among institutionalised individuals and in children attending group settings [46,47]. With regard to the transfer of genetic material, this appears to originate from the commensal streptococci *Streptococcus mitis* and *Streptococcus oralis*, present in the nasopharynx, which carry mutations in the *pbp* genes and transfer these to pneumococci, where they are subsequently incorporated by homologous recombination. The genetic alterations display a mosaic pattern, comprising sequences of variable size, and accumulate in the transpeptidases as a consequence of the selection pressure exerted by sustained β-lactam use, producing structural changes in the amino acids of PBP2x, PBP2b and PBP1a [13]. Additional determinants that modulate the level of resistance conferred by altered PBPs include alterations in the *murM* gene; notably, β-lactamase production has never been described in *S. pneumoniae* [13]. Macrolide resistance in pneumococci is attributed to the *erm*(B) gene, which encodes a methylase, thereby also conferring resistance to lincosamides and streptogramins (the MLS_B_ phenotype), whereas the *mef*(A) gene is involved in efflux-mediated resistance.

Macrolide resistance of the M phenotype in the pneumococcus is encoded by the *mef*(E)/*mel* and *mef*(A)/*msr*(D) gene pairs, expressed under the action of 14- and 15-carbon macrolides. The Mega element carries *mef*(E), encoding a proton-motive-force efflux pump, and *mel* [*msr*(D)], encoding an ABC-F ribosomal protection protein that displaces the macrolide from its ribosomal binding site; resistance requires both determinants, with Mel/Msr(D) contributing predominantly. Modification of the ribosomal target site for macrolides represents the most frequent mechanism by which macrolide resistance arises [48].

Two features of pneumococcal macrolide resistance are worth stating precisely, since they differ from the staphylococcal situation described in Section 3.2.7. The *mef*(E)/*mel* operon carried on the Mega element encodes two functionally distinct proteins that act together: Mef(E), a proton-motive-force efflux pump, and Mel (Msr(D)), an ABC-F ribosomal protection protein of the same family as the Sal-type and *optrA*/*poxtA* products discussed above. Neither determinant alone reproduces the full resistance phenotype. Expression of the operon is inducible by 14- and 15-membered macrolides, so that resistance may be underestimated by susceptibility testing performed without induction. The *erm*(B)-mediated MLS_B_ phenotype, by contrast, confers high-level, constitutive or inducible cross-resistance extending to lincosamides and streptogramin B, and the two determinants are increasingly found together on composite Tn*916*-family elements such as Tn*2010* [48].

Fluoroquinolone resistance in the pneumococcus is rare compared with that observed in staphylococci or in Gram-negative bacteria, but it does occur, either through efflux pumps, genomic mutations in *gyrA*, *parC* and *parE*, or the acquisition of resistance via plasmids [49].

Pneumococcal resistance is unusual among the pathogens discussed here in that it can be modified by vaccination. A systematic review and meta-regression of paediatric pneumococcal isolates collected worldwide between 2000 and 2020 found that the implementation of pneumococcal conjugate vaccines was followed by reductions in the prevalence of penicillin non-susceptibility, whereas no comparable reduction was observed for macrolide non-susceptibility in either vaccine-targeted or non-vaccine serotypes [50]. This dissociation is consistent with serotype replacement by non-vaccine lineages that retain macrolide resistance determinants, and it supports the medium-priority classification assigned to macrolide-resistant *S. pneumoniae* in the 2024 WHO Bacterial Priority Pathogens List [6,7]. Vaccination should therefore be regarded as complementary to, rather than a substitute for, stewardship and surveillance.

Resistance to classes other than the β-lactams and the macrolides receives less attention but is clinically relevant. Tetracycline resistance in the pneumococcus is conferred almost exclusively by *tet*(M), a ribosomal protection protein rather than a target-modifying enzyme, and *tet*(M) is carried on integrative and conjugative elements of the Tn*916* family; because *erm*(B) is frequently located on the same elements, macrolide and tetracycline resistance are commonly co-transferred and co-selected, and exposure to either class may select for both [45,48]. Resistance to trimethoprim–sulfamethoxazole arises from point mutation in *folA*, encoding dihydrofolate reductase, and from insertions in *folP*, encoding dihydropteroate synthase, rather than from acquisition of an alternative target; this distinguishes it from the *dfrA*-mediated mechanism described in staphylococci in Section 3.2.6 [45]. Multidrug resistance is concentrated in a small number of globally disseminated clones, of which serotype 19A of clonal complex 271, and within it sequence type 320, became the most prominent after the introduction of the seven-valent conjugate vaccine [45,48].

For pneumococcal infections caused by penicillin-resistant strains, the available therapeutic options include the antipneumococcal fluoroquinolones—levofloxacin, moxifloxacin, gatifloxacin—for pneumonia and bronchopneumonia, together with vancomycin and the third-generation cephalosporins ceftriaxone or cefotaxime. In the treatment of pneumococcal meningitis, vancomycin is recommended in combination with a cephalosporin until susceptibility results become available, with subsequent de-escalation to a third-generation cephalosporin. As alternatives, the combination of a cephalosporin with rifampicin or chloramphenicol appears equally effective [51]. Vancomycin remains effective against the pneumococcal strains isolated to date, but a tendency towards vancomycin tolerance has been observed in some strains, which could suggest the possible emergence of vancomycin resistance in the future [52,53,54].

### 3.4. Resistance in Enterococcus faecium

The position of *E. faecium* among the organisms considered here is a distinctive one. It is a gut commensal of modest intrinsic virulence that has become a leading cause of hospital-acquired bloodstream infection through the expansion of a hospital-adapted population, historically designated clonal complex 17 and now defined more precisely by genomic analysis as clade A1, which is separated by a substantial evolutionary distance from community-associated and animal-associated lineages and is characterised by ampicillin and fluoroquinolone resistance, by the insertion sequence IS*16* and by an enlarged accessory genome carrying determinants of colonisation and adhesion [55]. Its intrinsic resistance to cephalosporins, to clinically achievable concentrations of aminoglycosides given alone and to clindamycin, combined with an unusual readiness to acquire further determinants on plasmids and conjugative transposons, means that the therapeutic margin in enterococcal infection is narrow from the outset rather than as a consequence of acquired resistance alone [55,56].

*Enterococcus faecium* is among the pathogens under WHO surveillance, owing to an alarming increase in resistance, which may be multidrug-resistant (MDR) or XDR, and at times, even PDR.

*E. faecium* possesses six PBP genes, comprising class A—*ponA*, *pbpF*, *pbpZ*—and class B—*pbp5*, *pbpA*, *pbpB*—of which *pbp5* encodes a low-affinity class B transpeptidase that sustains peptidoglycan cross-linking, while the other PBPs are saturated by the antibiotic, thereby conferring intrinsic resistance to ampicillin and cephalosporins; the upstream genes *ftsW* and *psr*, located in the same operon, modulate PBP5 expression levels [57].

Ampicillin resistance is attributed to variations in PBP5, which confer two distinct types of resistance: high-level resistance (MIC above 128 μg/mL), PBP5-R, associated with hospital settings, and a community variant, PBP5-S, with an MIC below 64 μg/mL [58].

The enzymatic mechanism of ampicillin inactivation by β-lactamases has also been described, in *E. faecium* as well as in *E. faecalis*, through constitutive expression of the *blaZ* gene at a low level, conferring clinically a significant inoculum effect—a form of resistance that is overcome by the addition of a β-lactamase inhibitor such as sulbactam. A further mechanism of ampicillin resistance is attributed to the L,D-transpeptidase Ldt_fm_, which cross-links peptidoglycan via 3 → 3 bonds using a tetrapeptide donor, thereby bypassing the D,D-transpeptidation catalysed by the PBPs and conferring resistance to β-lactams other than the carbapenems, which inactivate L,D-transpeptidases [59].

Intrinsic resistance to cephalosporins is not fully understood at the molecular level; it is associated with the reduced affinity of PBP5 for the antibiotic, whose transpeptidase activity must be coupled with a glycosyltransferase in order for peptidoglycan to be synthesised. Experimentally, deletion of all class A genes produces a dissociation between resistance to cephalosporins and resistance to ampicillin, with resistance to the latter being maintained at the same MIC values [60,61].

Intrinsic resistance to cephalosporins is governed by two distinct signal transduction pathways. The first is the CroRS two-component system, in which the sensor histidine kinase CroS autophosphorylates in response to cell wall stress and transfers the phosphoryl group to its cognate response regulator CroR, which in turn modulates gene expression; deletion of *crores* in *E. faecalis* reduces the ceftriaxone MIC approximately 4000-fold [62,63]. A functionally equivalent CroRS system operates in *E. faecium*, where its deletion confers marked susceptibility to cephalosporins and bacitracin, and where it is required for the enhanced β-lactam resistance conferred by PBP5 overexpression [64]. The second pathway is not a two-component system but a eukaryote-like Ser/Thr kinase–phosphatase pair: the transmembrane PASTA-domain kinase IreK and its cognate PP2C-type phosphatase IreP, which act antagonistically. Deletion of *ireP* leaves IreK constitutively active and produces hyperresistance to ceftriaxone, at the cost of a substantial competitive fitness defect in the absence of antibiotic [65,66]. The orthologous kinase–phosphatase pair in *E. faecium* is designated Stk/StpA [67]. The two pathways are not independent: IreK phosphorylates CroS at a threonine residue required for its function, thereby positively influencing CroR-dependent gene expression [68].

Aminoglycoside resistance is associated with an enzymatic mechanism, as in staphylococci, through the synthesis of acetyltransferases, phosphotransferases or nucleotidyltransferases, and with mutations in ribosomal protein S12.

The chromosomally encoded 6′-acetyltransferase AAC(6′)-Ii of *E. faecium* inactivates netilmicin, tobramycin, sisomicin and kanamycin; some strains additionally possess APH(3′)-IIIa, a phosphotransferase conferring resistance to both kanamycin and amikacin [69]. Ribosomal target modification by the methyltransferase EfmM, which methylates C1404 of 16S rRNA, confers resistance to kanamycin and tobramycin [69]. Clinically, gentamicin and streptomycin retain synergistic activity with β-lactams because they escape these intrinsic enzymes. This synergy is nevertheless abolished by acquired high-level resistance—mediated by the bifunctional enzyme AAC(6′)-Ie-APH(2″)-Ia in the case of gentamicin, and by the adenylyltransferase ANT(6)-Ia or by ribosomal mutation in the case of streptomycin—which is why screening for high-level aminoglycoside resistance is required before combination therapy is selected [69].

With respect to daptomycin, resistance is associated with three genes: *liaF*, a component of the LiaFSR three-component system involved in the cell envelope stress response, increasing the MIC from 1 to 4 μg/mL and abolishing bactericidal activity; and the genes *gdpD*, encoding a glycerophosphoryl diester phosphodiesterase, and *cls*, encoding cardiolipin synthase, both of, which are involved in phospholipid metabolism [70,71].

Fluoroquinolone resistance is attributed to alterations in the *gyrA* and *parC* genes, as well as to efflux pumps. For quinupristin–dalfopristin, resistance results from ribosomal modifications, antibiotic efflux or enzymatic modification [72].

Resistance to oxazolidinones, specifically to linezolid, is attributed to alterations in the genes encoding the ribosomal target 23S rRNA, to mutations in the ribosomal proteins L3, L4, and L22, and to the action of the *cfr* gene, which is involved in the methylation of A2503—this gene has been identified in strains of *E. coli* and staphylococci and is readily transmissible between species via the mobile element IS*256*. With respect to dalfopristin/quinupristin, *E. faecium* acquires resistance through the acetyltransferases Vat(D) and Vat(E), which inactivate dalfopristin, and through the action of the lactonases Vgb(A) and Vgb(B), which enzymatically cleave streptogramin B (quinupristin) [73]. Further mechanisms in strains with the MLS_B_ phenotype involve the presence of the *ermB* gene, which affects the activity of quinupristin; the efflux pumps *msrC* [74] and mutations in the *eatA* gene [75].

Resistance to tetracyclines may be attributed to efflux of the antibiotic, encoded by the plasmid-borne genes *tet*(K) and *tet*(L), or to protection of the ribosomal target site. Chromosomal resistance to doxycycline and minocycline is associated with the genes *tet*(O), *tet*(M) and *tet*(S), transmitted by mobile elements of the Tn*916* transposon type. Resistance to doxycycline and minocycline is associated with the ribosomal protection genes *tet*(O), *tet*(M) and *tet*(S), which are typically carried on conjugative transposons of the Tn*916* family and become chromosomally integrated after transfer. With respect to tigecycline, resistance may arise through ribosomal mutations, mutation of the ribosomal protein S10, and deletion of the *tet*(M) [76], and, in European isolates, through upregulation of plasmid-encoded *tet*(L) and *tet*(M) [56], with a prevalence of 3.5% in Europe, compared with 0.3% in the Americas and 1.3% in Asia [77].

High-level vancomycin resistance in *E. faecium* is conferred principally by the plasmid-mediated *vanA* and *vanB* gene complexes, and the global expansion of this phenotype has been attributed largely to the hospital-adapted clonal cluster 17 (CC17) genogroup; the capacity of *E. faecium* to transfer resistance elements across the One Health spectrum was one of the considerations underlying its high-priority classification by WHO [6]. In the EU/EEA, the estimated incidence of bloodstream infection caused by vancomycin-resistant *E. faecium* has increased since 2019, in contrast to the declining incidence of MRSA bloodstream infection over the same period [12].

Epidemiologically, vancomycin resistance is the marker that defines the problem. WHO retained vancomycin-resistant *E. faecium* in the high-priority category of the 2024 Bacterial Priority Pathogens List, where it received the highest overall score of any Gram-positive organism assessed [6,7]. In the EU/EEA, the estimated incidence of bloodstream infection caused by vancomycin-resistant *E. faecium* has risen since 2019 while the incidence of MRSA bloodstream infection has fallen over the same interval, with the highest resistance levels reported from southern, central and eastern Europe [12]. The divergence of the two trajectories within a single surveillance system is informative: the infection-prevention measures that have reduced MRSA transmission have not had an equivalent effect on vancomycin-resistant enterococci, whose reservoir is the gastrointestinal tract and whose transmission is sustained by prolonged asymptomatic colonisation and by environmental persistence rather than by direct contact alone [55]. Resistance in this species is therefore not simply a pharmacological problem but a problem of colonisation pressure, and surveillance confined to invasive isolates will register it late.

*E. faecium* is an extremely adaptable organism, and currently, therapy is limited to the glycopeptides, cyclic lipopeptides, oxazolidinones and glycylcyclines.

Each of these options carries a specific constraint, and the constraints are cumulative. Daptomycin is bactericidal but requires doses above those licensed for staphylococcal infection when used against enterococci, and non-susceptibility emerging during treatment has been documented repeatedly, mediated by the LiaFSR cell-envelope stress response and by alterations in membrane phospholipid metabolism [31,56]. Linezolid is bacteriostatic and is compromised by the transferable determinants *optrA*, *poxtA* and *cfr*, which are not detected by molecular panels restricted to 23S rRNA mutations and which may be co-selected by agents of unrelated classes, including phenicols used in veterinary practice [29,56]. Tigecycline achieves low serum concentrations and is not recommended as monotherapy in bloodstream infection. Among the lipoglycopeptides, activity against *vanA*-type strains differs between individual agents and should not be inferred from activity against MRSA [55]. Taken together, these constraints make susceptibility-guided therapy, early source control and the containment of colonisation more consequential in enterococcal infection than in either of the other two organisms considered in this review.

### 3.5. Therapeutic Options and Future Prospects

Antibiotic resistance in the Gram-positive cocci discussed here necessitates the use of alternative antibiotics, among them the oxazolidinones, daptomycin, the new glycopeptides—dalbavancin, telavancin, oritavancin—with firm indications regarding the site of infection in relation to their pharmacological properties, tigecycline, and ceftaroline (for staphylococci). The use of antibiotics must be combined with source control (removal of devices, surgical drainage of abscesses, necrotic debris, and so on) and with antimicrobial stewardship, in order to prevent the injudicious use of therapy and to avoid the emergence of resistance to reserve agents, or of new resistance mechanisms arising from the selection pressure exerted by sustained use of a given antibiotic class. Combination therapy with synergistically acting antibiotics should be used in systemic infections, endovascular infections and biofilm-associated infections.

Table 3 is restricted to agents with reported activity against Gram-positive cocci; its licensed entries overlap in part with the broader tabulation in [2], from which the Gram-negative entries have been omitted. Three groups are distinguished: agents licensed for clinical use; agents that have been evaluated clinically but whose licensure is confined to particular countries or to a restricted route of administration; and candidates that have not been evaluated in humans. The grouping is intended to prevent the inference, which the earlier arrangement permitted, that a compound described as active against MRSA is thereby available as a therapeutic option.

The distinction between licensed agents and preclinical candidates matters when the prospects for Gram-positive infections are assessed. Ceftobiprole and dalbavancin have now been examined in adequately powered randomised trials in complicated *S. aureus* bacteraemia, and in both the comparison with existing standards was one of non-inferiority rather than superiority [79,89]; the practical advantages of these agents therefore lie in dosing convenience, in the avoidance of prolonged intravenous access, and in the availability of alternatives when toxicity or resistance limits established regimens, rather than in demonstrably better cure rates. Teixobactin, the malacidins, odilorhabdins and DCAP, by contrast, illustrate the renewed productivity of culture-independent and previously unexplored microbial sources, but all remain preclinical [91,92,95,96]. At a system level, the constraint is the size and the innovation content of the pipeline: in the most recent WHO analysis, the number of antibacterial agents in clinical development fell from 97 in 2023 to 90 in 2025 (50 traditional agents and 40 non-traditional approaches such as bacteriophages, antibodies and microbiome-modulating agents), and only 15 met the WHO criteria for innovation [98].

### 3.6. Limitations

Several constraints qualify the conclusions drawn here. The first concerns the design. The search was documented and reproducible, but screening was not conducted in duplicate with formal adjudication, no protocol was registered, and no instrument was applied to appraise the quality of individual sources; the selection of evidence therefore reflects the authors’ judgement of relevance and cannot be assumed exhaustive. Restricting retrieval to publications in English is likely to have excluded relevant national surveillance and clinical literature, and this loss is not randomly distributed, since several of the regions in which resistance in Gram-positive cocci is most prevalent publish substantially in other languages. Reliance on published sources also leaves the review exposed to publication bias, most consequentially in the therapeutic literature, where unsuccessful preclinical candidates and negative trials are reported less often than positive findings.

The surveillance evidence carries limitations that no review design can correct. GLASS aggregates data from countries whose laboratory capacity differs substantially, and roughly half of the reporting countries lack systems capable of generating fully reliable data [1]; global and regional figures should therefore be read as indications of magnitude rather than as precise values. Comparisons between surveillance systems are further constrained by differences in case definition, in the denominator reported—the proportion of resistant isolates among those tested is not equivalent to a population-based incidence, and the two are not interchangeable—and in susceptibility-testing standards, since CLSI and EUCAST breakpoints differ and both have been revised during the period covered. Passive laboratory-based surveillance also over-represents hospitalised and severely ill patients, so resistance proportions derived from blood cultures need not describe the organisms circulating in the community.

Finally, the therapeutic section is a synthesis of published evidence and not a comparative effectiveness assessment. The trials cited were not designed to be compared with one another, they differ in population and endpoint, and most were powered for non-inferiority rather than superiority. The discovery-stage agents discussed have not been evaluated in humans, and activity demonstrated in vitro or in animal models remains a weak predictor of clinical benefit.

Two further constraints follow from the search itself and were identified during peer review. The core Boolean query combined four concept blocks with the AND operator and was consequently specific rather than sensitive; records addressing only one aspect of the field—the epidemiology of a single organism, or a mechanism described without reference to therapeutic development—were not returned by that query and entered the review, when they did so, through the parallel free-text searches, the institutional sources or the reference lists of retrieved articles. Retrieval was therefore not exhaustive, and the synthesis should be read as an explicitly documented but incomplete sampling of a very large literature rather than as a comprehensive one.

## 4. Conclusions

Resistance in Gram-positive cocci remains a moving target rather than a solved problem. Global surveillance indicates that more than one in four *S. aureus* bloodstream isolates is methicillin-resistant, with marked regional heterogeneity, while European data show that the incidence of MRSA bloodstream infection can be reduced when infection prevention and stewardship are applied consistently, even as vancomycin-resistant *E. faecium* increases over the same period. The mechanistic picture has also become more complex than the classical *mecA*–PBP2a paradigm, encompassing *mec* homologues that escape routine molecular confirmation, transferable oxazolidinone resistance determinants, and the presence in staphylococci of resistance gene cassettes of a type characteristic of Gram-negative organisms, whose route of acquisition has not yet been established experimentally. Because the therapeutic pipeline is neither large nor markedly innovative, the measures that are likely to determine outcomes in the short term are those already available: rapid microbiological diagnosis including detection of resistance mechanisms, antimicrobial stewardship, infection prevention and control, and vaccination where an effective vaccine exists, together with equitable access to the agents that remain effective, since the newer options are also the most expensive and are least available in the settings where resistance is highest. The gaps identified in this review also indicate where effort is most needed: surveillance harmonised in case definition, denominator and breakpoint standard and reported by organism rather than in aggregate; multicentre studies and genomic epidemiology capable of tracking the determinants that routine confirmatory testing does not detect; and laboratory capacity sufficient to generate such data in the regions currently least represented in global reporting. By restricting attention to three Gram-positive cocci, this review makes visible a set of problems that a pan-bacterial account necessarily compresses: determinants that routine confirmatory testing does not detect, divergent regional trajectories within the same organism group, and a therapeutic repertoire whose most recent additions have demonstrated non-inferiority rather than superiority. Because the synthesis presented here was structured but not systematic, these conclusions are descriptive: they summarise published surveillance and mechanistic data and characterise the current therapeutic repertoire, rather than establishing causal relationships between any single intervention and the trends observed.

## Figures and Tables

**Table 1:** Methodological features of the present structured narrative review compared with those required of a systematic review reported according to PRISMA 2020.

Methodological Element	Systematic Review (PRISMA 2020)	Present Structured Narrative Review
Protocol and registration	Prospective protocol, publicly registered	No protocol prepared or registered
Research question	Single, narrowly specified question	Three related questions spanning epidemiology, resistance mechanisms and therapeutics
Search strategy	Exhaustive and sensitivity-oriented; fully reproducible	Explicit, dated and documented; specificity-oriented, complemented by institutional sources and reference-list screening
Screening	Independent duplicate screening with formal adjudication of disagreement	Titles and abstracts screened by both authors
Data extraction	Piloted, structured extraction instrument	Extraction by both authors without a structured instrument
Risk-of-bias appraisal	Mandatory; instrument-based	Not undertaken
Synthesis	Quantitative where the data permit (meta-analysis)	Qualitative and interpretative; no pooling of results
Certainty of evidence	Formally graded (e.g., GRADE)	Not graded
Flow diagram	Required element of PRISMA-compliant reporting	Used as a reporting template only (Figure 1); no claim of compliance

**Table 2:** Comparative surveillance indicators for the three Gram-positive cocci considered in this review.

Indicator	*S. aureus*	*S. pneumoniae*	*E. faecium*
Share of blood-culture isolates worldwide, 2023 (GLASS) [1]	21.9%	1.4%	Not reported separately in [1]
Defining resistance marker	Methicillin resistance (MRSA)	Reduced susceptibility to penicillin (oxacillin used as surrogate)	Vancomycin resistance (VRE)
Global resistance proportion, 2023 (GLASS) [1]	MRSA 27.1%	Oxacillin 17.8%; penicillin 5.2%; ceftriaxone 0.7%; cefotaxime 0.8%	Not reported in [1] as cited here
Highest regional value cited	50.3% MRSA, Eastern Mediterranean Region [1]	Not reported by region in [1] as cited here	Highest levels in southern, central and eastern Europe [12]
EU/EEA incidence of bloodstream infection, 2024 [12]	MRSA 4.48 per 100,000 population (country range 0.55–13.63)	Not reported in [12] as cited here	Increased relative to the 2019 baseline (absolute value not cited here)
EU/EEA trend, 2019–2024 [12]	Significant decrease (−20.4%); already below the 2030 EU target of 4.79 per 100,000	Not reported in [12] as cited here	Increase
Principal burden and affected population [5]	Sepsis; approximately one million deaths worldwide in 2019, reported from 135 countries, predominantly in patients over 15 years	Deaths predominantly in children under 5 years; lower respiratory tract infection	Hospital-acquired bloodstream infection, predominantly in immunocompromised and long-stay patients
WHO Bacterial Priority Pathogens List 2024 [6,7]	High-priority (MRSA); overall score 59%	Medium-priority (macrolide-resistant)	High priority (vancomycin-resistant); overall score 69%, the highest among Gram-positive organisms
Modifiable by vaccination	No licensed vaccine	Yes—pneumococcal conjugate vaccines reduce penicillin non-susceptibility but not macrolide non-susceptibility [13]	No licensed vaccine

**Table 3:** Therapeutic options for infections caused by multidrug-resistant Gram-positive cocci.

Agent/Class	Developmental Status	Reported Activity	Selected Supporting Evidence
Ceftaroline, ceftobiprole—fifth-generation cephalosporins	Licensed	Active against MRSA, VRSA; resistant strains also occur	In a phase 3, double-blind, randomised trial in 390 patients with complicated *S. aureus* bacteraemia, ceftobiprole was non-inferior to daptomycin with respect to overall treatment success [78,79].
Tedizolid—oxazolidinone	Licensed	Active against Gram-positive pathogens causing acute bacterial skin and skin-structure infections, including MRSA	[80,81]
Besifloxacin, delafloxacin, ozenoxacin	Licensed, topical only; licensed, systemic; licensed, topical only	Active against MRSA, *S. pneumoniae*	Delafloxacin in vitro activity was retained against *S. aureus* isolates non-susceptible or resistant to methicillin, vancomycin, daptomycin or linezolid. Besifloxacin showed bactericidal activity in vitro against staphylococci, *S. pneumoniae* and *H. influenzae* and has been evaluated in bacterial conjunctivitis; ozenoxacin has been reviewed for topical cutaneous use. Both are formulated for topical administration only and are not systemic options in invasive infection [82,83,84,85].
Lascufloxacin, levonadifloxacin	Licensed in selected countries only	Active against Gram-positive pathogens causing acute bacterial skin and skin-structure infections, respiratory tract infection	In use in Japan and India [86,87]
Omadacycline	Licensed; pathogen-specific clinical outcome data limited	Active against MRSA, VRE, *S. pneumoniae*	Documented in a microbiological and preclinical review; the entry rests on in vitro activity rather than on pathogen-specific clinical outcome data [88].
Dalbavancin, telavancin, oritavancin	Licensed	Active against MRSA, *E. faecalis*	In the DOTS randomised clinical trial (*n* = 200), dalbavancin was not superior to standard therapy on the day-70 desirability-of-outcome ranking, although the non-inferiority criterion for clinical efficacy was met [89]. Reviewed as a therapeutic option in selected periprosthetic joint infection [90].
Teixobactin (produced by *Eleftheria terrae*)	Preclinical	Active against MRSA	Discovered by screening uncultured soil bacteria; binds lipid II and lipid III, and no resistant mutants of *S. aureus* were obtained under the reported laboratory conditions [91].
Malacidins (natural product of the soil microbiome)	Preclinical	Active against MRSA	Calcium-dependent lipopeptides identified by culture-independent metagenomic screening; sterilised MRSA skin infection in an animal wound model [92].
Antimicrobial peptides (antimicrobial, anti-adhesion and antibiofilm activity)	Preclinical	Active against Gram-positive. Synergistic action with β-lactams, macrolides, fluoroquinolones	Reported to act synergistically with β-lactams, macrolides and fluoroquinolones, and to possess anti-adhesion and antibiofilm properties. A structurally heterogeneous class; activity varies between individual peptides [93].
Dodecyl deoxy glycosides	Preclinical	Active against Gram-positive bacteria by targeting membrane lipids	Membrane-lipid-targeting compounds discussed among approaches to overcoming resistance in Gram-positive bacteria [94].
DCAP—leads to bacterial membrane depolarisation and cell lysis	Preclinical	Active against Gram-positive, Gram-negative	Membrane-active small molecule that dissipates the transmembrane potential and increases membrane permeability, including in slow-growing bacteria [95].
Odilorhabdins—natural peptides produced by *Xenorhabdus nematophila*	Preclinical	Active against Gram-positive, Gram-negative	Bind a previously unexploited site on the small ribosomal subunit and induce miscoding; active in animal infection models [96].
Bacteriophage therapy	Preclinical for the indication cited	SATA-8505—active against *S. aureus* biofilm	Investigated in combination with antibiotics against *S. aureus* biofilms; the sequence in which phage and antibiotic were administered affected the outcome [97].

MRSA, methicillin-resistant *Staphylococcus aureus*; VRE, vancomycin-resistant enterococci; VRSA, vancomycin-resistant *Staphylococcus aureus*. Agents in the lower part of the table are investigational and have not been evaluated in humans; they are not available as therapeutic options. Licensure status and approved indications differ between regulatory jurisdictions and should be confirmed against the relevant national summary of product characteristics before any clinical use.

## Data Availability

No new data were created or analyzed in this study. Data sharing is not applicable to this article.
